# Superhydrophobic functionalized cellulosic paper by copper hydroxide nanorods for oils purification

**DOI:** 10.1038/s41598-021-95784-z

**Published:** 2021-08-10

**Authors:** Ahmed S. Belal, Jehan El Nady, Azza Shokry, Shaker Ebrahim, Moataz Soliman, Marwa Khalil

**Affiliations:** 1 Materials Science Department, Institute of Graduate Studies and Research, Alexandria University, P.O. Box 832, Alexandria, Egypt; 2 Electronic Materials Department, Advanced Technology and New Materials Research Institute, City of Scientific Research and Technological Applications (SRTA-City), New Borg El-Arab City, P.O. Box 21934, Alexandria, Egypt; 3 Nanotechnology and Composite Materials Department, Institute of New Materials and Advanced Technology, City of Scientific Research and Technological Applications (SRTA-City), New Borg El Arab City, P.O. Box 21934, Alexandria, Egypt

**Keywords:** Materials science, Nanoscience and technology

## Abstract

Oily water contamination has been sighted as one of the most global environmental pollution. Herein, copper hydroxide nanorods layer was constructed onto cellulosic filter paper surface cured with polydopamine, Ag nanoparticles, and Cu NPs through immersion method. This work has been aimed to produce a superhydrophobic and superoleophilic cellulosic filter paper. The structure, crystalline, and morphological properties of these modified cellulosic filter paper were investigated. Scanning electron microscope images confirmed that the modified surface was rougher compared with the pristine surface. The contact angle measurement confirmed the hydrophobic nature of these modified surfaces with a water contact angle of 169.7°. The absorption capacity was 8.2 g/g for diesel oil and the separation efficiency was higher than 99%. It was noted that the flux in the case of low viscosity solvent as n-hexane was 9663.5 Lm^−2^ h^−1^, while for the viscous oil as diesel was 1452.7 Lm^−2^ h^−1^.

## Introduction

The crude oils extracted from oil wells are not pure but are mixed with various liquids and solid matters such as dust, fine sand, sludge, clay, water with dissolved salts, oxides, hydroxides, sulfides, etc. The separation of these impurities is difficult to perform by just one method and the techniques of separation can be based on mechanical, thermal, chemical, or electrical character. These methods are slow, expensive, and inefficient^1–3^. There are porous materials such as sponges, foams, and textiles used to purify the oils from water. However, these materials are suffered from low capacity and rejection efficiency. Moreover, the recycling or reuse of these materials is difficult and time-consuming^4–6^. Consequently, the development of effective oil/water separation cost-effective, eco-friendly, and recyclable superhydrophobic nanomaterials with high efficiency and flux are in high demand^7,8^.

Superhydrophobic materials have been used for oil/water separation as metal^9^, fabric^6,10,11^ sponge^12,13^, paper^14^, etc. cellulosic filter paper has been received concern due to its porous structure made of microfibers and low cost. However, only a few reports of the superhydrophobic cellulosic filter paper including hydrogel-coated^15^, dip-coating^16^, and PDVB-PDMS decorated^17^ were applied in the emulsion separation. Developing green and low-cost materials by facile preparation approach for efficient separation is instantly required^18,19^.

This work is aimed to functionalize the cellulosic filter paper top surface with Cu(OH)_2_ nanostructures. The modification on this cellulosic filter paper is carried out by coating n-dodecanethiol to reduce the surface energy. In addition, we concern with both oil/water separation technologies based on the filtration and absorption methods. The different parameters such as separation efficiency, flux, and oil absorption capacity of this modified cellulosic paper filter for chloroform, dichloromethane, toluene, n-hexane, diesel, sunflower oil, olive oil, waste engine oil, and diesel oil are evaluated and compared.

## Materials and methods

### Materials

Cellulosic filter paper composed from high-quality cotton liners, which have been treated to achieve a minimum alpha cellulose content of 98% was bought from German (Whatman style filter paper is made from cellulose and is used for general filtration) and dopamine hydrochloride was obtained from Sunny Pharmaceutical Company. Ammonium hydroxide (25%) was obtained from Sigma. Silver nitrate (99.8%), sodium hydroxide (96%), and dichloromethane were purchased from Merk. Glucose was received from El Nasr Pharmaceutical Chemical Company. Copper sulfate pentahydrate (99%) and ethanol were bought from Fisher Scientific. Potassium sodium tartrate (99%) ammonium persulfate (98%), chloroform, toluene, and n-hexane were brought out from BDH company. Dodecanethiol (98%,) was purchased from Across Company. Diesel, sunflower oil, and olive oil were obtained from the local market.

### Preparation of copper hydroxide nanorods

Firstly, the cellulosic filter paper was washed with a mixture of deionized water and ethanol and consequently, it was modified by in situ polymerization of dopamine hydrochloride by immersing the cellulosic paper in the prepared 2 mg/mL dopamine solution with pH 8.5 (adjusted with ammonia) for 24 h at 60 °C with continuous stirring. This modified cellulosic paper was washed with deionized water. This modified surface of the cellulosic paper was treated with 0.05 M of AgNO_3_ and 0.01 M glucose with sustaining pH to 8.0 for 6 h. Finally, this modified cellulosic paper was washed with deionized water and dried at room temperature**.**

The Cu NPs layer onto Ag NPs modified cellulosic paper was dipped in a mixture of 80 mL Fehling B reagent solution. Moreover, the copper hydroxide layer was produced by immersing the last Cu /Ag NPs modified cellulosic paper in 1 M NaOH and 0.05 M ammonium persulfate for 30 min. The surface was also rinsed and washed with deionized water and dried at ambient temperature for 24 h. In the final step, the modified cellulosic surface was functionalized with an ethanolic solution of 0.01 M n-dodecanethiol for 30 min.

### Characterization techniques

The structural formed layers onto the cellulosic filter paper were characterized by Fourier transform infrared (FTIR) spectrophotometer (Spectrum BX 11- Perkin Elmer). The FTIR spectra were measured in the range from 4000 to 400 cm^−1^. Each layer was grinded with KBr powder and this powder was pressed to form a translucent pellet.

Copper hydroxide was characterized by Raman spectra which was recorded using a microscope equipped with triple monochromatic combined with a peltier cooled charge couple device detector system (Senterra Bruker). The spectra were acquired in the back-scattering geometry while the 532 nm line of an Argon laser source was focused on the sample for excitation at a power of 2 mW. The phonon frequencies were obtained by fitting Lorentzian line shapes to the experimental peaks after background subtraction.

The absorption spectrum of the suspension solution of PD/Ag/Cu and PD/Cu was recorded with a UV–visible spectrophotometer (Evolution 300, Thermoscientifc, USA). The measurement was performed in quartz cuvettes using H2O as a solvent. The light source wavelengths were scanned in the range of 200 to 600 nm.

The XRD analysis of the copper hydroxide formed onto the cellulosic filter paper surface was evaluated by Bruker- AXS D8 Discover. The Bragge’s angle (2θ) in the range from 5 to 80 degrees was varied. The X-ray source of Cu target was used at 30 kV and 30 mA. The morphology of the modified cellulosic filter paper was examined by SEM attached with energy dispersive X-ray (EDX) (“JEOL JSM 6360LA”, Japan). The modified cellulosic filter paper layers were sputtered with Au film. Cu(OH)_2_ nanostructure was confirmed with high-resolution transmission electron microscope (HRTEM) attached with selected area electron diffraction (SAED) (JEOL, JEM-2100 LaB6).

The hydrophilicity and hydrophobicity of the modified cellulosic filter paper were evaluated by contact angle measurements using Rame-hart, France. The average of the 5 measurements at different positions was recorded.

### The performance of modified cellulosic filter paper for oils and organic solvents removal

The absorption process was measured for different viscous oils (diesel engine oil, waste engine oil, olive oil, and sunflower oil) and organic solvents such as n-hexane, dichloromethane, chloroform, and toluene. The absorption capacity (Q in g/g) was calculated by the following equation^4^:

1 $$ {\varvec{Q}} = \frac{{{\varvec{m}}_{{\varvec{e}}} - {\varvec{m}}_{{\varvec{o}}} }}{{{\varvec{m}}_{{\varvec{o}}} }} $$

where ***m***_**o**_ is the initial weight of the modified cellulosic filter paper and ***m***_**e**_ is the weight of the modified cellulosic filter paper after immersing in oils or organic solvents and water.

The modified surface of cellulosic filter paper was used as a filter for oil (organic solvents)/water separation. Oils or organic solvents/water mixtures with the volume ratio of 1:1 (50 mL of dyed oil with blue ink and 50 mL of dyed water with green ink) was used. This modified cellulosic filter paper was fixed at a separation system and serving as a separation membrane. The reused of the modified cellulosic filter paper was repeated for more than 100 cycles. The efficiency of separation of oils or organic solvents/water mixtures was estimated from the following Eq. ^20^:

2 $$ {\varvec{Separation}}\;{\varvec{Efficiency}} \left( \% \right) = \frac{{{\varvec{m}}_{1} }}{{{\varvec{m}}_{2} }}*100 $$

where ***m***_**1**_ and ***m***_**2**_ are the weight of the collected oil and the initial weight of oil, respectively. The flux of oils (organic solvents) in L m^−2^ h^−1^ (V) was calculated from the volume of the oils or organic solvents from the following equation ^20^:

3 $$ {\varvec{Flux}} = \frac{{\varvec{V}}}{{{\varvec{St}}}} $$

where ***S*** is the surface area of cellulosic filter paper and ***t*** is the separation time**.**

## Results and discussion

### Structural of the modified surface of cellulosic filter paper

**Figure 1:**
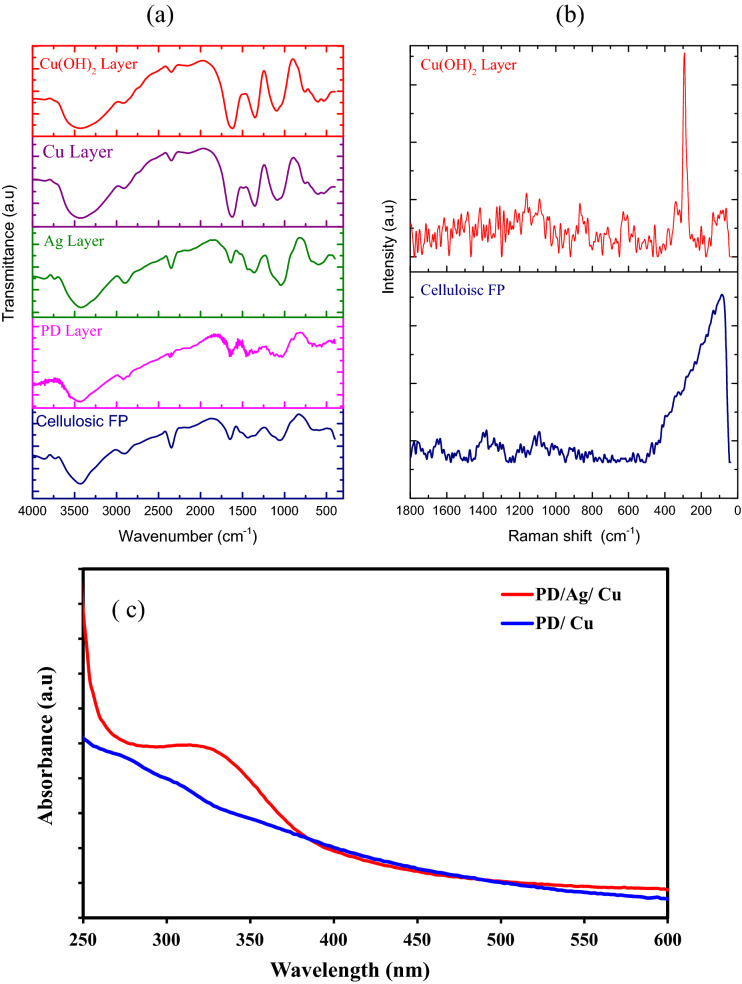
FTIR (**a**) Raman (**b**) spectra of cellulosic filter paper modified with different layers**,** (**c**) UV–Vis of PD/Ag/Cu and PD/Cu.

The cellulosic feature is confirmed by FTIR spectra for the pristine and modified cellulosic filter paper as shown in Fig. 1a. The broad absorption band at 3400 cm^−1^ is matched with O–H stretching vibrations arising from hydroxyl and hydrogen-bonded hydroxyl groups^21^. The peak at 1635 cm^−1^ assigns to O–H bending vibration of the adsorbed water^22,23^**.** The bands of anti-symmetrical C-O and C–O–C stretching vibrations of cellulose appear at 1132 and 1025 cm^−1^, respectively^24^**.** The peak at 1430 cm^−1^ is related to C–H scissoring motion of cellulose. The absorption bands at 2900 cm^−1^ and 1340 cm^−1^ are assigned to C–H stretching and bending vibrations, respectively. The polymerization of the polydopamine layer onto cellulosic filter paper surface is confirmed by indole or indoline moieties and hydroxyl groups that appear at 1637 cm^−1^ and 3300–3550 cm^−1^, respectively^21,25,26^. The bands of the infrared spectrum of cellulosic filter paper coated with PD/Ag NPs layers are slightly shifted. The formation of Ag NPs layer does not affect the surface chemical structure of polydopamine^27^**.** The peak at 3550 cm^−1^ attributed to the stretching vibrations of -OH and N–H groups in the polydopamine is broadened due to the formation of the hydrogen bond between PD and Ag NPs layers. The peaks at 1604 cm^−1^, 1512 cm^−1^, and 1367 cm^−1^ are recognized to the stretching vibrations of –C=C–, –C=N and C–N–C functional groups associated with indoline (or) indole structure present polydopamine^28^.The good stability of the hierarchical structure of the celluloisc/PD/Ag fabrics arising from the chemical bonds between Ag NPs and polydopamine^26^. Messersmith et al. reported the formation of multifunctional polymer coatings through dip-coating of some materials in the solution of dopamine. They used dopamine self-polymerization to form thin films onto inorganic and organic materials. Secondary coatings can be added to create a variety of ad-layers^29^. The formation of Cu NPs in the presence of a catalyst Ag NPs onto PD surface is realized and Ag NPs prevent the formation of CuO NPs. It was found that the rate of Cu NPs drastically increases in the presence of Ag NPs, while without the Ag NPs only a coating of the reaction vessel surface is observed^30^'.

**Figure 2:**
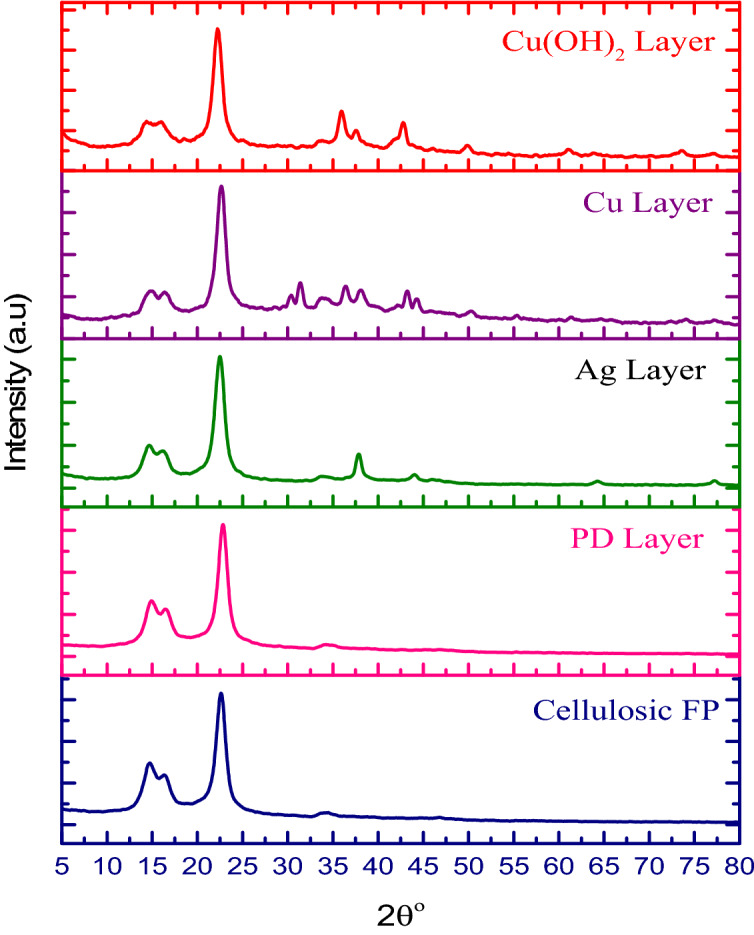
XRD patterns of cellulosic filter paper modified with different layers.

The new peak at 1477 cm^−1^ related to C=O groups is assured to the formation of Cu NPs on cellulosic fabric filter paper and is due to cellulose oxidation during the reduction of Cu ions. Also, the peak detected at 1614 cm^−1^ is indicated the stretching vibration of C=O bond in the cellulosic chain^**.**^ The broad peak at 3200–3600 cm^−1^ relates to OH bond stretching vibrations. This confirms that the absorption of Cu NPs onto the cellulosic fabric has occurred through the physical attachment^31^**.** The final layer of Cu(OH)_2_ functionalized cellulosic filter paper produced from the oxidation of Cu NPs by NaOH and ammonium persulfate is emphasized by the IR vibrations spectrum shown in Fig. 2. C–O stretching vibrations at 938 cm^−1^ can be assigned to the corresponding metal cation (Cu^2+^) in Cu(OH)_2_ which appears at 933 cm^−1^ for pure Cu(OH)_2_. The band at 750 cm^−1^ is attributed to the bending vibrations of H bonded OH groups. In addition, Cu–O stretching and Cu–O–H bending vibrations have appeared at low frequencies at 610 cm^−1^^32–34^.

The prepared copper hydroxide samples were also characterized by Raman spectroscopy. Raman spectroscopy is a successful apparatus for looking at the surface structure of cellulosic filter paper, so it is reasonable for investigating the surface layer of copper hydroxide NPs. Figure 1b shows the Raman spectra of Cu (OH)_2_ on the cellulosic filter paper. The peak at 453 cm^−1^ was assigned to a Cu–O vibrational mode, while the peak at 865 cm^−1^ was assigned to Cu–O–H vibrations a strong and sharp band at 293 cm^−1^^35,36^.

The absorption peak of the formation of Cu NPs in presence of PD appears after a long time with low intensity as shown in Fig. 1c. On the other hand, a high absorption spectrum at 324 nm of Cu NPs appears due to the catalytic effect of Ag NPs^37,38^. Ag NPs as the catalytic activation seeds can catalyze Cu NPs to deposit on the surface of fabrics. In the initial stage of the reaction, Cu ions in the copper sulfate solution are preferentially reacted with Ag NPs due to the strong catalytic activity of Ag NPs^37,39^. As a result, Cu NPs are dispersed uniformly to cover the surface of cellulosic fibers.

Figure 2 depicts XRD patterns of the pristine and modified cellulosic filter paper surfaces to determine their crystal type and crystallinity. The pure surface of cellulosic filter paper appears characteristic cellulose patterns at 14.7°, 16.7°, 22.7°, and 34.5°, respectively qualified to the diffraction planes of (1–10), (110), (200), and (004). It is observed that the layer of PD has no effect on the crystal structure of cellulosic filter paper owing to the construction of a thin and amorphous layer of PD. The existence of Ag NPs layer is proved onto modified cellulosic filter paper/PD by the formed of the cube phase for Ag NPs at 37.7°, 38.1°, 44.1°, 64.6°, and 77.5° which are assigned to (111), (111), (200), (220), and (311) planes, respectively^27,40^**.** No diffraction peaks for Ag oxide or halides were observed, indicating that elemental Ag was present on the surface of polydopamine-coated cellulosic filter paper.

The sharp diffraction peaks at 43.7°, 44.5°, 50.7°, and 74.3° concern Cu NPs and they are corresponded to (111), (111), (200), and (220) planes of face centered-cube structure, respectively. The crystalline phase of the Cu(OH)_2_ nanostructures is shown in Fig. 2. The diffraction peaks noted at 16.6°, 23.8°, 34.1°, 39.7^o^, and 53.5° are assigned to the (020), (021), (002), (130) and (150) planes, respectively, of orthorhombic structure and this confirms of formation high crystalline Cu(OH)_2_^41^**.**

### Morphological properties

**Figure 3:**
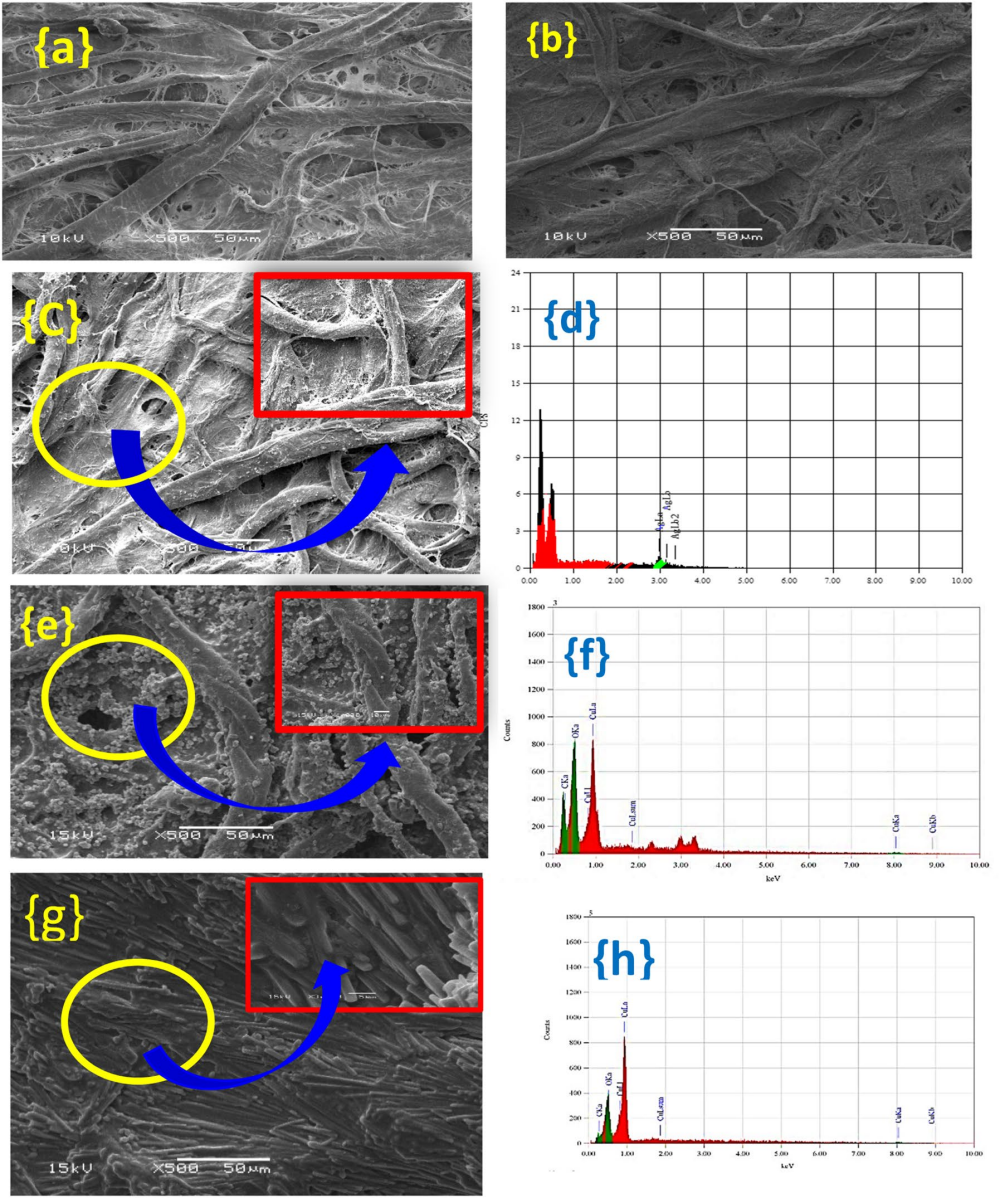
SEM images of modified cellulosic FP surfaces with different layers (**a**) Pure cellulosic FP, (**b**) PD layer, (**c**) Ag NPs layer, (**e**) Cu NPs layer, and (**g**) Cu(OH)_2_ nanorods layer. EDX analysis of (**d**) Ag layer, (**f**) Cu layer and (**h**) Cu(OH)_2_ layer.

The SEM images and EDX of each layer synthesized on the cellulosic filter paper are illustrated in Fig. 3. The surface of cellulosic fiber has a fibers structure in the microscale range with inhomogeneous diameters from 1 to 30 μm to form a network structure that seem too tight as displayed in Fig. 3a. According to the SEM image of the PD layer shown in Fig. 3b, the PD layer is transparent, uniform, and bright coated onto the cellulosic filter paper substrate. In addition, the morphology of the modified surface of cellulosic filter paper with PD/Ag depicted in Fig. 3C has been precipitated with irregular fine shapes. These particles are continuous and connected with each other. Ag NPs with about 100 nm are observed on a rough PD coating. The existence of the Ag NPs layer on the surface of modified FP is emphasized by the green lines presented in the EDX spectrum of Fig. 3d.

Cu NPs coating layer formed and shown in Fig. 3e onto the modified cellulosic filter paper with PD/Ag NPs substrate has a rough structure. It is observed that the well-covered and large Cu particles formed in the range of 0.5 μm on the surface of cellulosic filter paper. Figure 3f displays the existence of Cu particles in the EDX spectrum. Through a typical oxidizing reaction to form copper hydroxide, the Cu particles become and transformed into nanorods as shown in Fig. 3g. The presence of the copper and oxygen % is 32.5213 to 67.4787%, in the modified cellulosic filter paper as depicted in Fig. 3h and it is noticed that the amount of copper is half of the amount of oxygen that proves the formation of copper hydroxide.

**Figure 4:**
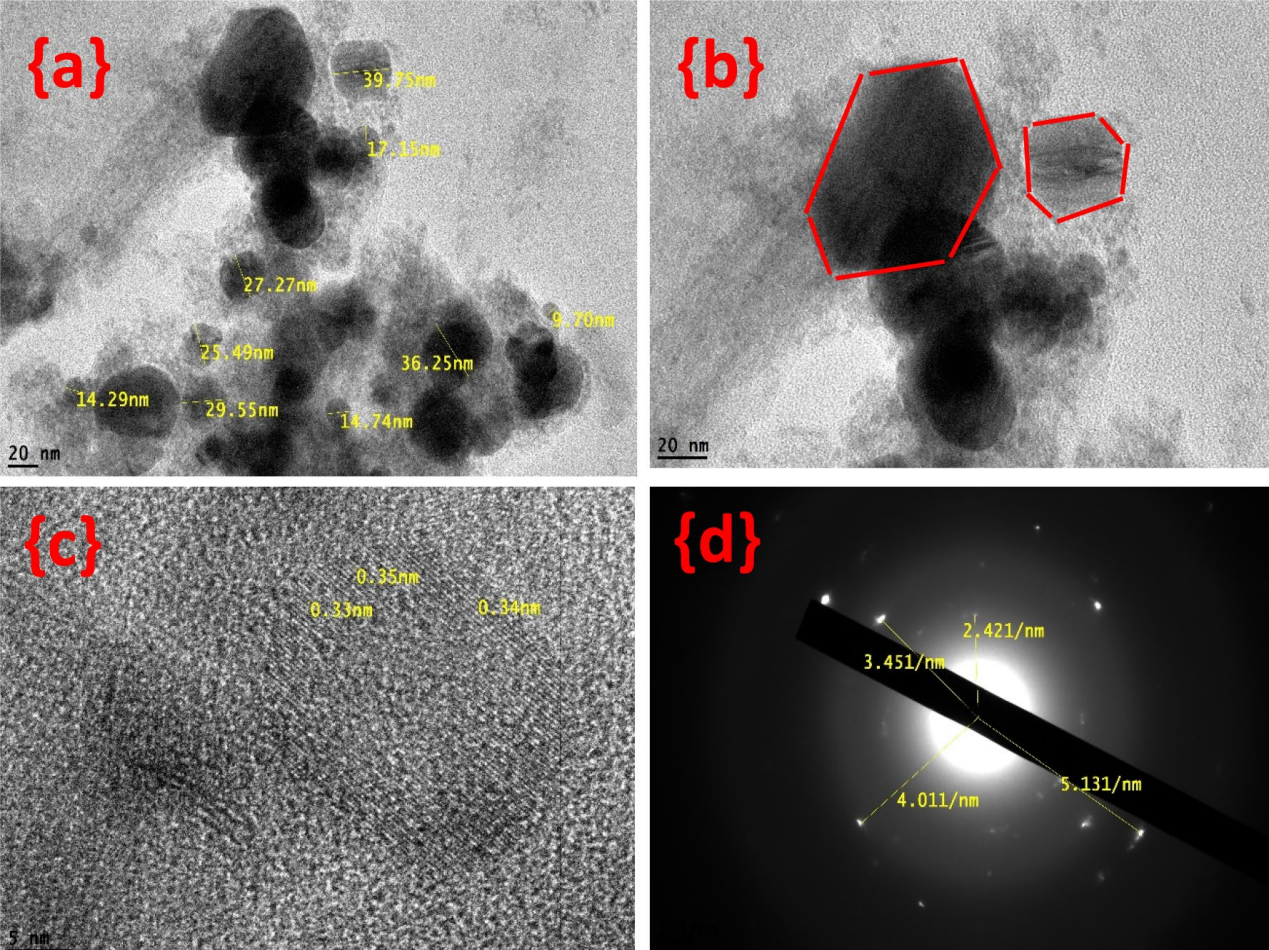
HRTEM images of copper hydroxide at different magnifications (**a**, **b**) and the SAED (**c**, **d**).

As shown in the HRTEM images in Fig. 4, the prepared copper hydroxide is a uniform hexagonal with mean diameters of approximately 9–39 nm. At high magnification, the lattice space is observed to be 2 Ǻ. SAED pattern can be used to identify the crystal structures. There are clear diffraction rings with white spots indicating polycrystalline nanoparticles with a 5.6 Ǻ lattice distance**.**

### Hydrophobicity of the modified surface of cellulosic filter paper

Generally, the cellulosic filter paper surface is superhydrophilic due to its structure^42^. The Cu(OH)_2_ nanorods grown on the surfaces of the cellulosic filter paper enhance surface roughness and the n-dodecanthiol coating reduces the surface energy to increase the superhydrophobic property. The modified cellulosic filter paper is designed for oils (organic solvents) /water separation. This material will be directly in contact with mixture oil (organic solvents) and water during the service.

**Figure 5:**
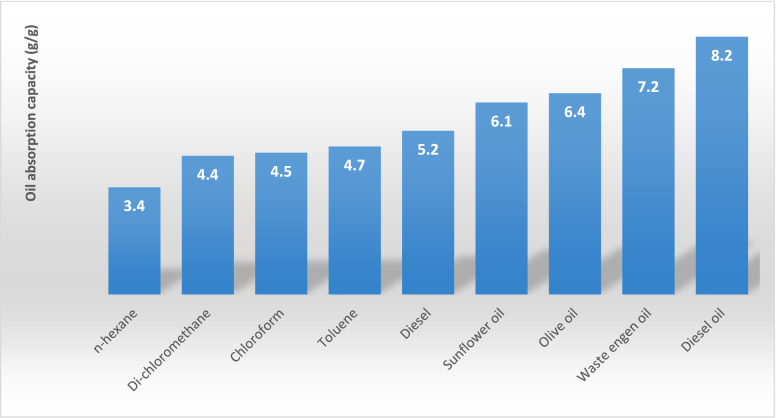
Absorption capacities of the modified surface of cellulosic filter papers in different oils and organic solvents.

The three parameters that control the performance of the application of the superhydrophobic modified cellulosic filter papers in oil and water separation are the absorption capacity of the oil, separation efficiency, and water flux. The fabricated superhydrophobic functionalized cellulosic filter paper significantly absorbs the different organic solvents and some oils. Figure 5. illustrates the different range of oils and organic solvents absorption for the capacities of the modified Cu(OH)_2_ nanorods grown on the surfaces of the cellulosic filter paper. These modified filter papers have high oil and organic solvent absorption capacities. This can be elucidated based on the Cu(OH)_2_ nanorods grown onto the cellulosic filter paper is highly lipophilic and is easily wetted with the viscous oils. The oil absorption capacities values are 3.4, 4.4, 4.5, 4.7, 5.2, 6.1, 6.4, 7.2, and 8.2 (g/g) for n-hexane, dichloromethane, chloroform, toluene, diesel, sunflower oil, olive oil, waste engine oil, and diesel oil, respectively**.**

**Figure 6:**
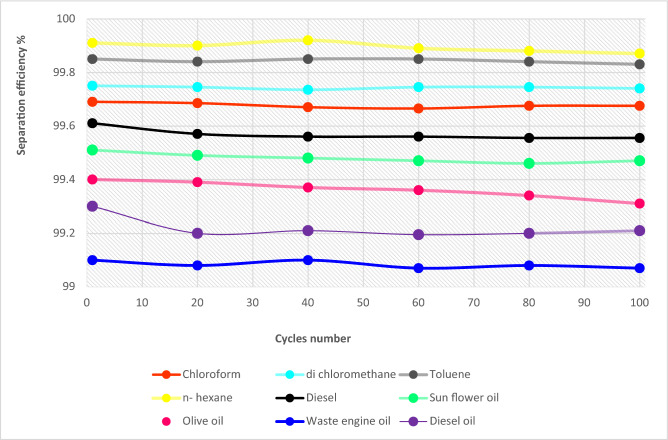
Separation efficiency of modified cellulosic FP membranes for different oils and solvents.

**Figure 7:**
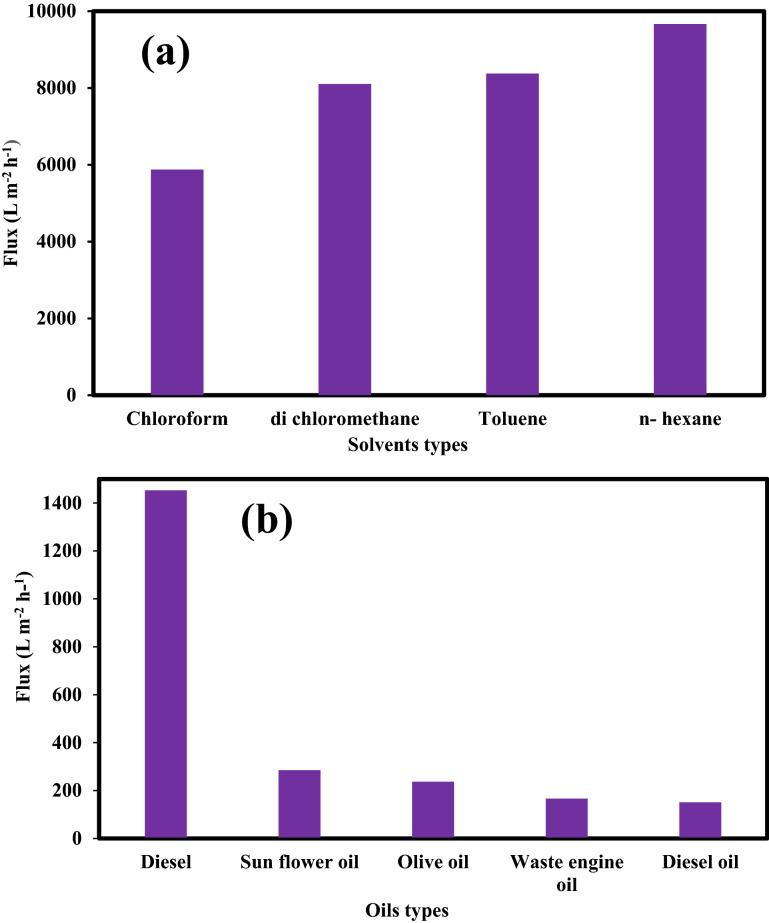
Flux of the modified surface cellulosic filter papers (**a**) low viscous and (**b**) high viscous organic solvents.

The separation efficiency and the oil flux of oil or organic solvents are measured and calculated and the separation efficiency using the modified cellulosic filter paper is higher than 99% for the oil/water mixtures. From Fig. 6, it is observed that the separation efficiency of the modified filter paper is higher than 99% and maintains this value after 100 cycles. The values of these efficiencies are found to be 99.68%, 99.74%, 99.84%, 99.9%, 99.57%, 99.5%, 99.36%,99.1%, and 99.2% for chloroform, dichloromethane, toluene, n-hexane, diesel, sunflower oil, olive oil, waste engine oil and diesel oil, respectively. In addition, Fig. 7a. presents high values of the permeate flux values for the different oils and organic solvents through the Cu(OH)_2_ nanorods modified filter paper. This is cleared for the low viscous solvents and the permeate flux values are5876, 8105.2, 8375.7, and 9663.5 Lm^−2^ h^−1^ for chloroform, dichloromethane, toluene, and n-hexane, respectively. In addition, the oil flux values for the viscous oils such as diesel, sunflower, olive, waste engine, and diesel oils are 1452.7, 284.7, 236.8, 166.3, and 150.8 Lm^−2^ h^−1^ respectively as observed in Fig. 7b. The value of flux mainly depends on the viscosity of different oils. To measure the water content in the oil, we used Karl Fisher coulometric titration. Karl Fischer analysis for water content can yield highly accurate results. It was detected the water content in the oil before and after the oil–water separation process for n-hexane as low viscous oil and diesel as high viscous oil. We notice that the water contents for n-hexane and diesel are 0.01% and 0.02%, respectively. After the separation process, the water contents are to be 0.02% and 0.03% for n-hexane and diesel, respectively.

## Conclusions

Converting the surface of the cellulosic paper from superhydrophilic to superhydrophobic using immersion technique was investigated by deposition of Cu(OH)_2_ nanorods then coating this rough surface with n-dodecanthiol. The water contact angle of the modified surface of the cellulosic paper is 169.7^ο^. The superphydrophobic modified cellulosic papers possess good stability in both water and oil, which indicates that they can be used as a stable oil/water separation material. The modified surfaces of cellulosic papers had a high absorption capacity for diesel oil is 8.2 (g/g). The separation efficiency for different oil/water mixtures was higher than 99% and the flux of this modified cellulosic paper for low viscous n-hexane and high viscous diesel oil were 9663.5 and 150.8 L m^−2^ h^−1^, respectively. It was detected that these modified surfaces of cellulosic paper were stable for 100 cycles and one month.
